# Crises and Resilience at the Frontline—Public Health Facility Managers under Devolution in a Sub-County on the Kenyan Coast

**DOI:** 10.1371/journal.pone.0144768

**Published:** 2015-12-22

**Authors:** Mary Nyikuri, Benjamin Tsofa, Edwine Barasa, Philip Okoth, Sassy Molyneux

**Affiliations:** 1 Department of Health Systems and Research Ethics, KEMRI/Wellcome Trust Research Programme (KWTRP), P.O. Box 230–80108, Kilifi, Kenya; 2 Health Economics Unit, University of Cape Town, Observatory 7975, Cape Town, South Africa; 3 The Ethox Centre, Department of Public Health, University of Oxford, Old Road Campus, Headington, Oxford, OX3 7LF, United Kingdom; 4 The Centre for Clinical Vaccinology and Tropical Medicine, Nuffield Department of Medicine, University of Oxford, Old Road Campus, Headington, Oxford, OX3 7LF, United Kingdom; University of South Australia, AUSTRALIA

## Abstract

**Background:**

Public primary health care (PHC) facilities are for many individuals the first point of contact with the formal health care system. These facilities are managed by professional nurses or clinical officers who are recognised to play a key role in implementing health sector reforms and facilitating initiatives aimed at strengthening community involvement. Little in-depth research exists about the dimensions and challenges of these managers’ jobs, or on the impact of decentralisation on their roles and responsibilities. In this paper, we describe the roles and responsibilities of PHC managers–or ‘in-charges’ in Kenya, and their challenges and coping strategies, under accelerated devolution.

**Methods:**

The data presented in this paper is part of a wider set of activities aimed at understanding governance changes under devolution in Kenya, under the umbrella of a ‘learning site’. A learning site is a long term process of collaboration between health managers and researchers deciding together on key health system questions and interventions. Data were collected through seven formal in depth interviews and observations at four PHC facilities as well as eight in depth interviews and informal interactions with sub-county managers from June 2013 to July 2014. Drawing on the Aragon framework of organisation capacity we discuss the multiple accountabilities, daily routines, challenges and coping strategies among PHC facility managers.

**Results:**

PHC in-charges perform complex and diverse roles in a difficult environment with relatively little formal preparation. Their key concerns are lack of job clarity and preparedness, the difficulty of balancing multidirectional accountability responsibilities amidst significant resource shortages, and remuneration anxieties. We show that day-to-day management in an environment of resource constraints and uncertainty requires PHC in-charges who are resilient, reflective, and continuously able to learn and adapt. We highlight the importance of leadership development including the building of critical soft skills such as relationship building.

## Background

Public primary health care (PHC) facilities play a potentially valuable role in the implementation of primary health care in developing countries, and for many individuals are the first point of contact with the formal health care system [1–3]. However these facilities face significant challenges in levels of resources, quality of care, and accessibility to potential users [4]. Such challenges have been fuelled by wider problems of insufficient political prioritisation of health, structural adjustment policies, poor governance, and population growth [5]. Given the importance of primary health care for the achievement of Universal Health Coverage, the strengthening of public sector primary public facilities is a priority for many Low and Middle Income Countries. Facility strengthening requires an understanding of the priorities and concerns of those who work at the interface between health systems and communities, including facility staff and managers, through tracking how they are involved with and affected by policies and interventions as they unfold over time[6, 7].

One general approach to strengthening primary health care has been decentralization. Decentralization of the health system involves a variety of mechanisms to transfer fiscal, administrative, ownership and/or political authority for health service delivery from the central Ministry of Health to alternative institutions, or to intermediate or local levels ([8, 9]). Potential benefits of decentralisation include improved efficiency through greater cost consciousness and control at the local level, and better quality, transparency, accountability and legitimacy[8, 10]. Although evidence on the performance of decentralisation is relatively weak, partial and inconsistent, the data that is available suggest that quality improvements have often not materialised[8]. Challenges in practice have included insufficient transfer of decision-making power to local levels, lack of clarity in responsibilities of key players, and broader factors such as the prevailing political context, and inadequate access to financial resources [8, 9, 11].

The impact of decentralisation on PHC facility in-charges has received relatively little in-depth research attention, despite the key role that they play in implementing health sector reforms both as health care providers and as facilitators of change and of initiatives aimed at strengthening community involvement[12]. Health facility staff can be impacted upon by health sector reforms in unintended and sometimes damaging ways. Through research in Zambia, for example, Mogensen and Ngulube observed that frontline health providers were finding themselves in the centre of increasingly strained relations between the government and community members as a result of the introduction of user fees [13]. As they explained:

*Health workers experience that they deliver services which are compensated or reciprocated neither by their employers (the government)*, *since salaries are meagre and working conditions bad*, *nor from below*, *since patients’ contributions are not making a noticeable difference to the health workers’ living standards*. *In addition*, *users feel that due to the fees they pay (which do make a noticeable economic difference for them) they can make higher demands upon the health workers*. *The latter*, *however*, *only rarely have the resources with which to make any noticeable difference in quality of service*. *They therefore lose dignity in the eye of the ‘donors’ (the patients and the administrators who each pay them’)* p.24.

The managers of these facilities and health workers that work within them have been a particularly ‘neglected group in the system’, potentially playing a central role in the inter-connectivity between health administration, health workers and patients [14]. In this paper we describe the roles and responsibilities of PHC facility in-charges in Kenya, where there has been an accelerated process of political decentralisation in recent years.

### Devolution of Health Care Services in Kenya

Following the March 2013 election in Kenya, there was a shift from a centrally governed country with more than 80 districts to 47 semi-autonomous county governments [11]. Within the health sector, devolution meant the transfer of specific functions including service provision and ownership of health facilities to the county level, while the national Ministry of Health retained functions such as policy direction, regulations and standards. The transfer of power from central to county government was expected to take place over a three year period, and to be accompanied by other key changes for the health sector such as the merger of what was formerly two Ministries of Health (MoH), and the handing over of district level functions to the larger county government[15]. Another important change introduced at the same time was the official removal of all user fees at public primary level facilities and of maternity fees from all public hospitals.

**Fig 1:**
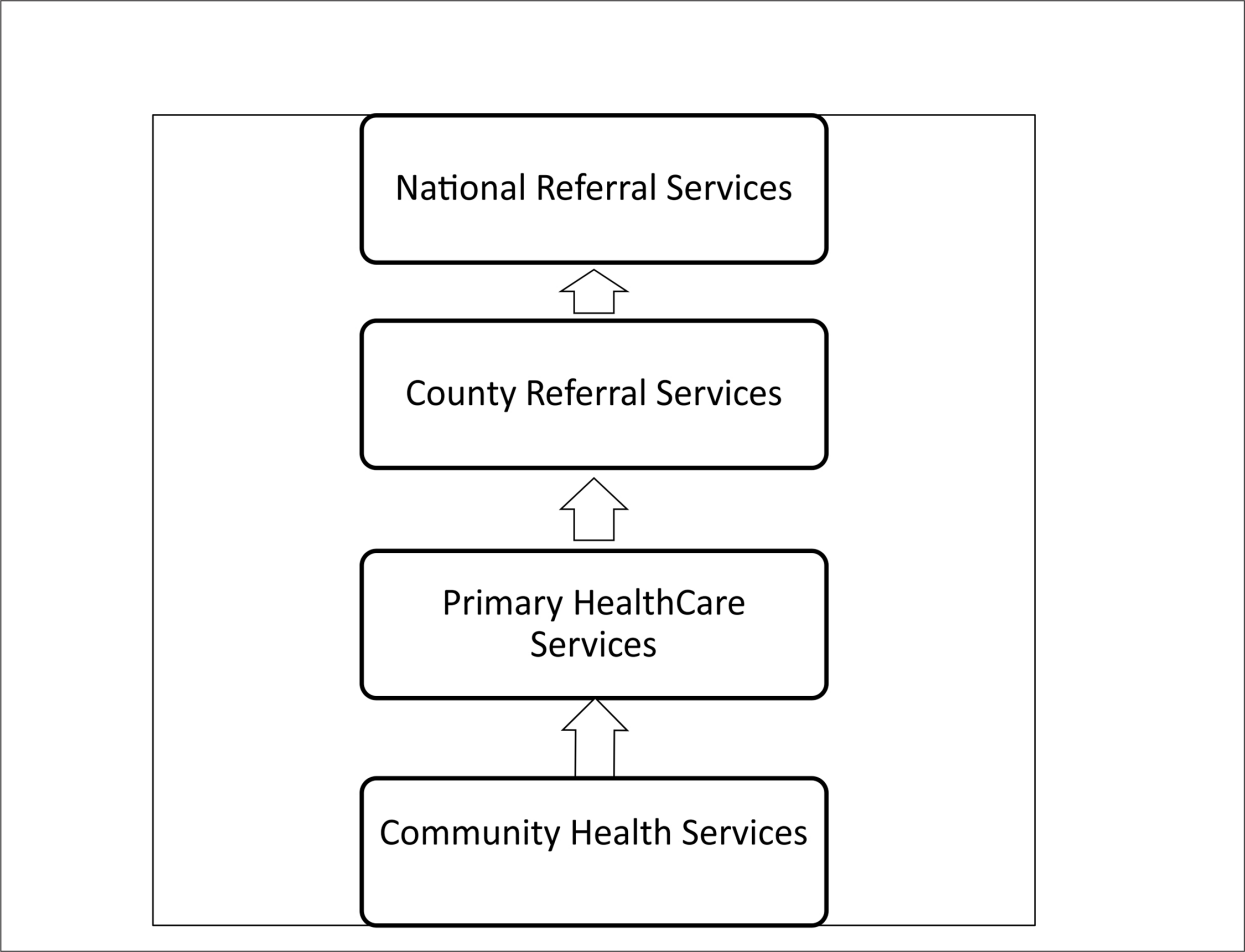
Organization of health services in Kenya under a devolved system.

Each county in Kenya can now organise its own health system governance structure. Many counties have adapted previous structures, introducing within their county department of health, a new County Health Management Team (CHMT) that oversees health issues in all sub-counties. Sub-counties tend to be managed by a sub-county health management team, or SCHMT, typically replacing what used to be a District Health Management Team (DHMT). The SCHMT provides support and management to all public and private health centres and dispensaries in their sub-county. These primary facilities are grouped under level two in the county health system hierarchy (Fig 1). At this level the focus is on health promotion and basic treatment, including simple diagnostic and short term in-patient services such as maternity care and short recuperative observations. Public PHC facilities are managed by either a nurse or a clinical officer commonly referred to as the “in-charge” or facility manager. These terms are used interchangeably in this paper.

**Fig 2:**
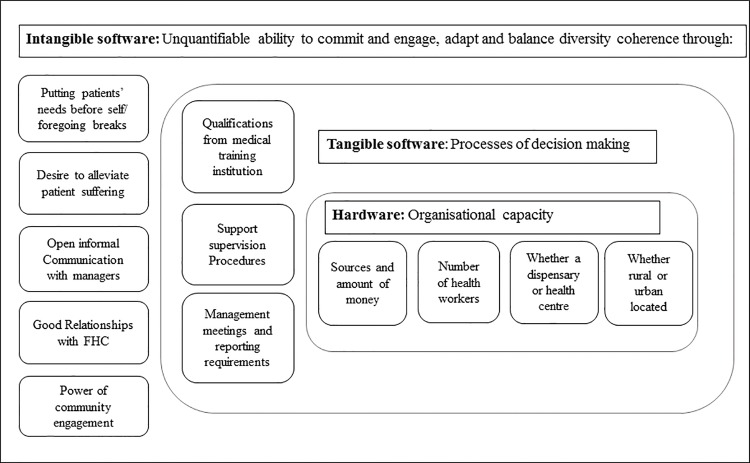
Organisational capacity adopted from Ortiz Aragon, 2010.

In examining the practices and routines of PHC facility in-charges operating in level 2 of the Kenyan system under devolution including their daily routines, the challenges they face, and the coping strategies they adopt, we contribute to a small but growing literature focusing on the micro-processes of governance at sub-national and local levels in LMICs. We also draw on the distinction made by Ortiz Aragon and others (Fig 2) between three interacting dimensions of organisational capacity: the *hardware* of infrastructure, technology and funding levels; the *tangible software* of knowledge, skills and processes of decision making; and the *intangible software* of relationships, communication practices, values and norms. The intangible features have been argued to be particularly important in shaping the behaviours of those working in an organisation and to underpin that organisations’ “power to perform” [16, 17].

## Methods

### Study setting

The data presented in this paper are part of a wider set of activities aimed at understanding governance changes under devolution in Kenya, conducted under the umbrella of a ‘learning site’. A learning site is a geographical area in which a long term process of collaborative research is implemented, involving health managers and researchers deciding together what the key health system questions and interventions are. The approach includes repeated interactions over long periods of time that built trust and familiarity, and that enable the invaluable tacit knowledge of health managers to be accessed. The approach was initiated by collaborators in South Africa because of its’ potential to overcome many of the challenges common in health systems research, including the difficulty of disentangling the effects of governance changes from those of parallel changes occurring in the health system, and the potential for opposition to the research if it is considered to be irrelevant or undermining by health system managers[6].

The Kenyan learning site is situated in Kilifi County, which in 2013 was estimated to have a population of 1. 2 million, 68% of whom live below the poverty line [18]. The county has 93 public and 110 private facilities with a nursing ratio of 37:100,000, and a doctor ratio of 1:137,500. Kilifi County covers seven constituencies, now sub-counties, but managed by three District Health Management Teams (now SCHMT). Health facilities are unevenly distributed across the county, with most located along major roads. This has resulted in unequal distribution of basic amenities and services within the county. It has also hampered easy access to these services by more far-flung rural communities [19, 20].

We selected one of the three sub-counties as the focus for the primary public facility work. Of the two sub counties not included in this work, one has other KEMRI research activities being undertaken while the other was inaccessible due to ongoing road construction. It had one health centre (now has two) and 17 dispensaries all of which are now managed by a sub-county management team. On the basis of discussions with sub-county managers, we selected the only health centre and three dispensaries for more in-depth observational and interview work. The three dispensaries were purposively selected to allow for variety in distance from main roads and urban centres, number of staff, and range of services offered.

### Observations and interviews at facility and county level

We conducted formal and informal interviews and observations over a one year period (June 2013 to July 2014). MN spent a continuous four week period in each of the four facilities with return visits over the rest of the fieldwork period. During the observation period, she spent all day in the facility, observing daily routines and activities, and having informal conversations with all staff members and some patients. She also held formal tape-recorded interviews with eight individuals in facilities over that time, usually broken into two different sessions of one to three hours each.

Primary public facility work was complemented by non-participant observation of sub-county level managers in their daily activities over by MN and PO over the same period, including formal interviews with seven managers. MN, SM and BT all live in Kilifi County, and have regular interactions with several of the sub-county and senior county leaders. BT in particular has a key role in our research and understanding: as well as being a governance researcher, he was a previous senior manager in the district himself, is a technical health advisor at county and national levels, and is a prominent member of the local community.

### Data management and analysis

Digital recordings of interviews were transcribed, managed and coded in NVIVO 10 using a framework analysis approach [21]. Coded data were organized into charts according to major themes based on the study objectives and issues emerging from interviews and reflective practice sessions. To support our analysis and shared learning, we organised a series of formal ‘reflective practice sessions’ of between three and five hours among the research team and where possible managers. In these meetings we shared what we learnt in the field and from the wider literature and policy and practice interactions, and the implications for our work and findings.

### Ethical consideration

The empirical work was approved by ethics committees of the Kenya Medical Research Institute Ethical Review Committee (SSC 2205). Permission to conduct the research was also obtained from the Kilifi county department of health, and from the three sub-county medical officers of health. As part of the research process, prior to data collection, the research team met with all participants to discuss the nature of the study, the methods that would be used in the study including observations. Before observations, the research team requested for permission to attend and document meeting proceedings. Verbal informed consent was obtained for all observations, and written informed consent for all formal interviews, and these consent processes were approved by the ethics committees of Kenya Medical Research Institute (KEMRI) and the London School of Hygiene and Tropical Medicine. All data were anonymised, with access limited only to researchers. This paper was published with permission from the Director KEMRI.

## Results

Following an overview of the basic hardware of the four facilities, we describe the roles and responsibilities of PHC facility in-charges, and some of the key challenges they face. For each of the key challenges, we reflect on the extent to which these appeared to be caused by, or exacerbated by, the devolution process as it unfolded at national and county level.

### Describing the four study health facilities

The four health facilities were quite different from each other in terms of size, layout and infrastructure (Table 1). The health centre is relatively large and well-staffed, while the smaller dispensaries vary significantly in size with between six and 15 rooms, and between one and three technical staff members. The health centre has housing for staff, serves a relatively large population of 30 to 40 thousand and offers 24 hour service, while dispensaries only offer services during the day to a population of 10 to 15 thousand. All facilities had running piped water, although at one dispensary water had been disconnected two months prior to the time of MN’s visit due to an unpaid bill. Two dispensaries are based in a rural area, and one in a peri-urban area, while the health centre is in an urban setting.

**Table 1:** Research Facilities description.

Facility characteristics	HC1	Disp1	Disp2	Disp3
Facility size (number of rooms excluding toilets, verandas and staff housing)	26 (3 wards)	7(6 in use, 1 abandoned)	6 with 3 semi-permanent rooms	15 spacious new rooms (6 old planned for renovation to be a maternity wing
No of technical staff	17 including 2 Medical officers	3 (2 nurses and a chew)	3 (2 nurses and 1 HTC counsellor)	1 (a Nurse)
No. of support staff	6	2	4	2 and a volunteer
Official opening hours	24hrs	08.00–17.00	08.00–17.00	08.00–17.00
Lab available?	Yes	No	Yes	No
Emergency referral system	Reliable Public transport	Reliable Public transport	Unreliable Public transport	Un available
Urban or rural	Urban	Peri-urban	Rural	Rural
Workload	300–350 per day	Data not available	Data not available	60
Functioning electricity supply	Yes	Yes	Yes	Yes
Running piped water	Yes	Yes	Disconnected over unpaid bill	Yes
Staff housing on site?	Yes	No	No	No
Functional telephone for facility use?	No(uses personal mobile)	Yes (though uses personal mobile)	No (uses personal mobile)	No (uses personal mobile)
Fridge and fridge thermometer	Yes	Yes	Yes	Yes
Source of funding	*HSSF, Kenya Government & *OBA	HSSF, Kenya Government & OBA	HSSF, Kenya Government & OBA	HSSF, Kenya Government, donors, user fees

*HSSF—Health Sector Services Fund

*OBA—Output Based Aid

All four facilities were initiated as community projects but have been taken over by the government. In addition to government support, they have received support for infrastructural development from well-wishers, donors and political actors.

### What does a PHC in-charge do?

There is no clearly laid out job-description or terms of reference for PHC facility in-charges that we could identify. However in-charges were described by sub-county managers as being the most senior clinical staff member in the facility, performing clinical and managerial duties in both health centres and especially dispensaries. As one Sub-county manager reported, in-charges should be:

*…Responsible for overall leadership*, *the governance bit*, *the management bit*, *all those things; they need to be aware of that*. *They need to be accountable and own that facility*. *They need to feel like that facility is their own*. *So it’s like their home in which they need to make sure that everything has been done appropriately*… First sub-county manager sub-county two

However, they described their roles in terms of what they did, being responsible for ensuring coverage and delivery of services, including through planning patient flow in a way that all users are served by the available staff. They are responsible for staffing, budgets, drugs, equipment, infrastructure, data and records, including as appropriate making orders, monitoring stocks and quality, and reporting on needs to their sub-county line-managers. For casual workers, they are responsible together with facility committee members for hiring, setting salaries, and oversight. For technical staff, they can only make recommendations to the sub-county on the nature and type of staffing they need. In order to plan appropriately, in-charges should develop together with key local stakeholders an annual work plan (AOP) which outlines the activities and resources needed to achieve facility targets. As the accounting officer for the facility and government employees, they are ultimately responsible for spending and accounting for budgets through a quarterly report to the sub-county accountant.

*…So basically they are supposed to plan as a small unit*, *then they incorporate the planning with the facility committee…*Female Sub-County Manager Sub-county one

**Fig 3:**
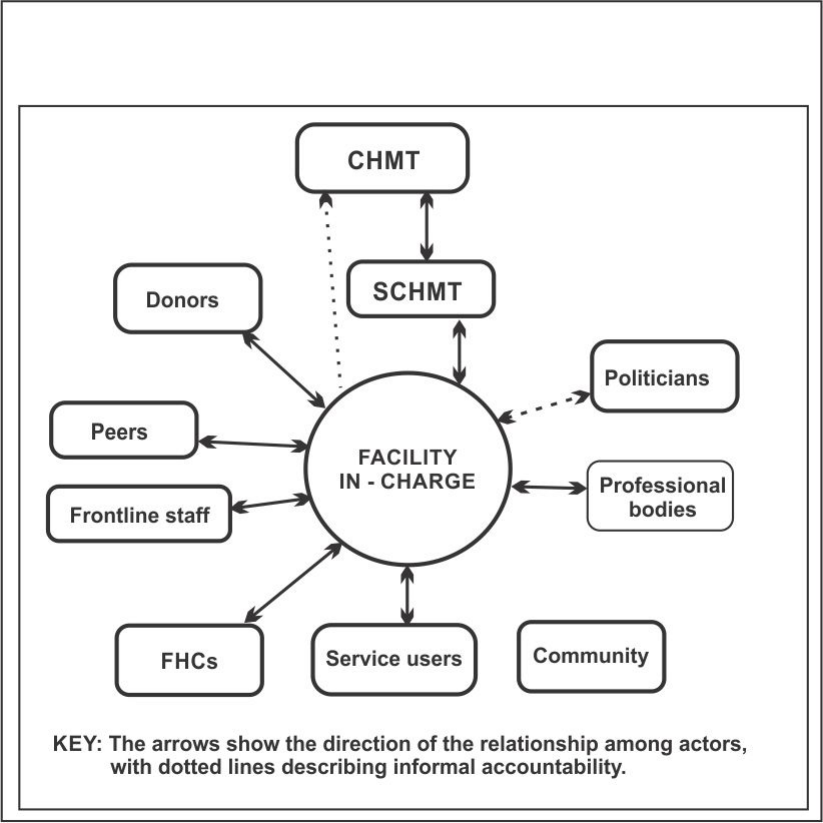
Network of actors that a facility in-charge is accountable to.

From observations and document review, it emerged that the health facility in-charge has various answerability requirements, upwards to supervisors, donors, politicians, and professional associations, horizontally to colleagues, and downwards to health facility management committees (Fig 3). With some actors there should be regular formal meetings (for example quarterly supervision from managers, and quarterly meetings with HFMCs), while with others meetings are less regular or formal. There are two key changes in these networks since devolution. Firstly, what were the previous district level mangers became sub-county level managers, as noted above and secondly, a new political cadre emerged in elected or nominated Members of the County Assembly (MCAs). Some MCAs have taken an active interest in the functioning of their local health facilities.

PHC facility in-charges also spent significant periods of time away from facilities attending to planned and unplanned meetings and trainings.

*…There are those scheduled meetings and trainings and then there are instances (unscheduled) whereby we tell them to close the facilities if at all this message is very important for them to know and to go and implement*…Female Sub-county manager in Sub county one

Interviews revealed that the amount of time spent on different activities varied significantly over time, with reporting requirements being particularly heavy at the end of the month and quarter, as discussed below

### In-charges’ challenges and coping strategies

Key challenges for in-charges were identified iteratively over the course of the fieldwork, and could variously be related to the three dimensions of capacity noted above—hardware, tangible software and intangible software–as is illustrated in the following sections.

#### Lack of preparedness for and clarity in role

A general overall challenge faced by facility in-charges is lack of preparation for their roles. In terms of formal training, they are relatively well prepared to play their professional role of a clinical officer or a nurse through their training at the Kenya Medical Training College or University as described in their current training manual[22]. However they have little preparation for their many leadership and management roles.

*…What I have been hearing is that nursing officers*, *when they finish schooling… they are just being informed that ‘you are going to be posted as the in charge of this facility’*, *and from school you didn’t hear anything about management*… First female sub county manager in sub-county two

The four in-charges we interviewed had been in service for very different periods of time, ranging from one to seven years. However all remembered how ‘unprepared’ they were to manage facilities on their first posting, and how ‘shocked’ or ‘overwhelmed’ they were by the breadth of their roles.

*…a posting letter explains nothing except the facility where you are going*. *So they just give you drugs and then you go*. *And in fact me I didn’t know what I was expected to do*. *I thought I was coming here to do nursing but when I came here there were so many things to do…*A female PHC in-charge at facility two

A particular issue raised across our work was who the in-charge actually is in a facility. Specifically there was a debate about whether this should always be based on length and type of experience, or on the nature and level of formal training. Interviews with sub-county managers indicated that clinical officers were generally preferred over nurses for the position of in charge because of their training. This was fairly straight forward at the health centre we visited: the clinical officer was the overall manager while the nursing officer was in charge of service delivery, and they both agreed with that ‘as policy’. However at one of the larger dispensaries, there was tension between the nursing officer and clinical officer over who should be overall ‘in charge’. The nursing officer had been in place and functioning as the in-charge since the facility’s inception six years earlier, while the clinical officer had only been posted there three months before we visited the facility. The clinical officer described considerable tension resulting from the situation, which he did not know how to handle:

*…She is scared of powers being taken away- she is seeing me as a threat instead of as a colleague*, *yet powers belong to the facility…* A male deputy PHC in-charge at facility two

Through reflecting on these issues with managers and research colleagues who have worked with facility in-charges for many years, it was clear that problems around role clarity, level of preparedness for in-charges, and tensions between cadres regarding seniority have long been a feature of primary public facility life and have not been impacted upon in a meaningful way by devolution.

#### Amount of reporting

Many of the relationships that in-charges have are accountability relationships, in which actors are answerable to one another. These accountability relationships are horizontal to colleagues and peers, upwards to funders and managers, and downwards to communities (Fig 3).

An issue raised in relation to the range of actors that in-charges need to be accountable to was the amount of paper-filling and reporting that they have to do (Table 2), which was variously described as ‘overwhelming’, ‘repetitious’, ‘confusing’ ‘tedious’ and ‘distracting’. Reports to be submitted to government line-managers include monthly service reports such as Malaria, TB and HIV cases (broken down by age category), number of test kits used, bed net distribution, and ANC attendance. In-charges also submit several financial reporting forms, including expenses against budget lines for every purchase and payment made. Beyond being accountable to government line managers, all facilities also had some responsibilities to donors who were supporting with staff or infrastructure. For example three facilities were members of a Reproductive Health Output-Based Aid (OBA) voucher scheme and had to submit delivery reports to the donor every month. To fulfil accountability responsibilities to communities, in-charges not only meet regularly with Facility heath committees (FHCs), but also have to ensure black boards at facilities include monthly outcome indicators and details on collections and expenditures.

**Table 2:** Amount of reporting–some of forms to be filled.

Clinical forms to show performance	Financial manuals and forms	
MOH711: TB control data	Guidelines and reference documents	Receipt vouchers(F017)
MOH711A: Integrated RH, HIV/AIDS, Malaria, TB &Nutrition	Managing HSSF, an operations manual	Payment vouchers(F021)
MOH113:Nutrition Monthly reporting	Guidelines on financial management for HSSF	Travel Imprest form(F022)
MOH717:Service workload	Chart on accounts	Local Purchase Orders (LPO)
MOH718:Inpatient morbidity and mortality	Registers and books to be completed	Local Service Orders (LSO)
MOH729AF:CDRR for ARV and OI medicine	Memorandum vote book	Request for quotations
MOH730: Facility monthly ARV patient	Receipt book	Stock cards for all items in stores
MOH731: Comprehensive HIV/AIDS facility reporting form	Facility service register	Imprest warrants
MOH733B: Nutrition services summary tool	Cash book	Bank reconciliation forms (F030)
MOH734F:CDRR HIV nutrition commodity	Cheque book	Counter requisition and issue vouchers(S11)
MOH105:service delivery report	Cheque book register	Counter receipt vouchers(S13)
MOH515: Chew summary	Fixed asset register	Handover forms
MOH710: Vaccines and immunization	Imprest register	Monthly financial report forms (MFR)
	Consumables stock register	Monthly expenditure report forms(MER)
	Store register	Quarterly financial report forms (QFR)
	Receipt book register	Other items register
	Forms/vouchers	

All four in-charges described having to work on weekends or past official hours to complete reports, particularly at month and quarter ends when reports are due in. All reports were felt to be important by in-charges and sub-county managers, to indicate workload, facilitate resource allocation, and plan supportive supervision and mentorship and coaching:

*…reporting is very important*. *That will make those supervisors know that you are working because you cannot be seen to be working unless you produce that report*. *You don’t say that I saw… fifty patients yesterday… nobody will believe you that you saw those patients but if the books are there the names are there then somebody can say yes*, *this is work done…* A male PHC in charge at facility four

*…[It]*. *Can then be seen how heavily you are burdened; so that you can reinforce the place*, *with maybe more nurses or more drugs*, *or more facilities that the continuity of services is there*…. A female PHC in-charge at facility two

However a concern raised particularly by in-charges is that they rarely receive feedback on issues raised through reports. It was also repeatedly mentioned by both in-charges and their managers that it would be useful to simplify reporting requirements and reduce repetition across tools that the in-charges have to submit.

*…Some are repetitions… You report on things like thrice*. *From the immunization there’s a tool for immunization then in the workload it’s all there; there’s a tool in malaria and then in another tool comes giving a whole report of the same*, *so it’s ambiguous*! *Why don’t we write in one tool…*? Male PHC in-charge at facility one

Reporting challenges are largely with upward accountability, with financial reports in particular considered difficult and worrying given that in-charges can be held formally responsible for any anomalies. Further challenges are the conflicting requirements of donors and government managers in some cases as illustrated below. Challenges with accountability downwards were less regularly raised. Relationships with HFMCs were mixed across facilities, with some evidence that in-charges worked with facility committees to challenge more senior managers, as discussed in the next section. Relationships between in-charges and MCAs were mixed and evolving; also touched on in the next section.

**An illustration of how an in charge dealt with external actors.** From interview and document review, dispensary 3 began as a community self-help project to reduce maternal mortality in the area. A donor who was approached to support the facility demanded that the facility be named after him. After the said donor left and returned to his country, the community struggled to keep the clinic running. After approaching the GoK, the MoH took over and sent staff as well as drugs and commodities. Besides, the facility receives HSSF and all MoH support, and thus basically became a public health facility.

On a recent visit, the signage was white washed and promises of the donor name put up, but by use of delaying tactics, the donor left before this was done. The facility remained nameless for a week. When the in charge was asked how he deals with such challenges, he said:

…*If the government takes charge of a facility*, *it should be fully responsible for it*, *but it seems the government also needs these donors to continue but the donors give their own requirements and the government also has their own requirements so sometimes it differs*, *but if can you can spend USD0*.*05 to get USD 0*.*2*, *then why no*t…? Male PHC in-charge at facility four

The implication is that as a PHC in charge has to continuously reflect on the benefits of various accountability relationships and their cost and be able to learn, adapt to survive.

As with problems around role clarity and preparedness, these accountability burdens and issues were a feature of PHC facility life before recent devolution. However there are several areas in which there appears to be some change. Firstly, there is further demystification about what happens with data and reports, including what is held and acted upon at (sub-county level) and what is forwarded on to national level. Secondly, and relatedly, was a lack of clarity in roles and responsibilities between the county and sub-county level management teams, including in relation to support and oversight of in-charges. Thirdly is the addition of the new political cadre of often quite vocal MCAs into accountability networks.

#### Resource scarcity

Resource scarcity has long been a challenge for primary public facilities in Kenya, and was regularly noted and observed during our observation period. Significant challenges in hardware were observed in terms of financial resources and drugs more specifically. As will be shown, coping with the challenges required intangible software skills including careful communication and engagement with sub-county managers, facility committees, and patients. Firstly, in Kenya, health centres and dispensaries have always controlled relatively few resources. Until the recent change of government, the central Ministry of Health supplied facility infrastructure, qualified health staff, drugs and equipment, and provided money for operational expenses such as support staff, maintenance, allowances, fuel, and non-medical supplies. However operational expenses were often failing to reach facilities and so user fees were often being charged by facilities to cope with shortfalls.[23, 24] Recognition of these challenges contributed to the introduction in 2010 of an innovative national health financing intervention called the Health Sector Services Fund (HSSF). Through HSSF, a fixed level of funds is sent directly to facility accounts every quarter from central level to assist with facility operations (for dispensaries, approximately USD 340 and USD 1200 for health centres). Although the amount of money is small compared to facility budgets and there have been teething problems, there were impressive achievements across the country with HSSF in terms of ensuring that funds reach facilities, and that funds are being overseen and used in a way that strengthens transparency and community involvement[25]. Quality of care was also generally reported to have improved, particularly in dispensaries, but user fees above national policy levels continued to be charged.

Our observations and discussions revealed significant financial challenges since devolution. One major challenge was a gap in receiving HSSF funds in facilities for six months following the presidential decree. This was related to several key funders of the initiative disagreeing on how HSSF should function under devolution, including on whether funds should now be dispensed through counties. This major set-back for facilities was compounded by the public announcement in June 2013 that all user fees would be removed from facilities. Facility in-charges found themselves losing their funds from national level and from user fees at around the same time. A series of crises resulted at facilities, including accumulation of water and electricity bills (to the point of both being disconnected in one facility), inability to pay casual workers, and community members demanding free services that were not available.

Facility managers developed a range of strategies in an effort to keep facilities open and functioning. Drawing on their prior relationships with sub-county managers they began to re-introduce user fees, in some cases working together to challenge more senior managers as illustrated below. Each in charge developed his or her own approach to doing this, in all cases in discussion with HFMC members who in general were keen to support the continuation of services and could see the predicaments of the in-charges. One facility reintroduced a system loosely based on the previous national policy, another introduced higher fees, arguing that the previous national policy was a registration fee only and that other charges were need to cover for treatments and procedures. A third facility decided that USD 0.3 would be charged for all users regardless of age and in the final one, the donor-funded OBA scheme was made a requirement for accessing reproductive health services to boost facility resources. Over time, although funder differences continue, the HSSF funds, user fee and maternity fee loss compensation funds were once again channelled into facility bank accounts, and appeared to play a critical role in facility functioning. However, there were new complexities and lack of clarity in terms of allocation criteria as well as reporting requirements at county and national level. Several facilities resolved to retain user fees as a buffer against future delays and given continuous resource constraints anyway. OBA funds also continued to be much appreciated as an added source of funding for the whole facility, rather than as a means specifically to improve Reproductive Health services.

**Example of innovation on delayed funding.** On 1st June 2013, the President of Kenya announced the removal of user fees and maternity fees from primary health facilities. The Government was going to compensate the facilities through direct funding to their bank accounts. However, four months down the line, no funds had been transferred to facilities, leading to a cash crisis—casual workers and utility bills went unpaid, and outreaches could not be conducted. In the face of water and electricity disconnection, filthy facilities (with casual staff leaving) and imminent closure, one facility in charge turned to her facility committee for a solution. Together with her committee they agreed to re-introduce user fees until the Government released the promised money. Upon learning of this development from Members of the County Assembly, a senior county manager sought to know under whose power the in-charge had acted against a Presidential directive. This incident prompted a visit to the facility by a very high level county team, without informing or inviting sub-county level managers (previously and possibly still the direct supervisors of the facility in-charges). The facility committee stood their ground and supported the in-charge, and managed to convince senior county managers that services could not stall because of central government delays in funding. This innovation by one facility in charge with her committee became an official temporary solution for the whole county. **Source: Researchers reflective meeting 25_10_2013**

Secondly, drugs were noted to be scarce and although drug shortages are not new to facilities, devolution apparently exacerbated the situation for 5 months by creating a change in the drug procurement system in the county from a prepaid system to a post-paid system.

In the old system each gazetted facility had an account with the Kenya Medical Supply Agency (KEMSA) where they were allocated a certain amount of money for drug purchase. The facility in charge ordered according to facility consumption and supplies were deducted from the overall balance. KEMSA used to supply directly to facilities according to their orders.

In the new system, funds allocated for drugs and pharmaceuticals are channelled through the county and the county pharmacist has to do quantification for the whole county, and then place an order to KEMSA. KEMSA makes the deliveries to the county and the county pharmacist has to ensure the supplies get to individual facilities as per their order. In the new system, supply depends on payments and late payments by the county results in delayed delivery causing regular stock outs. We also observed that when stocks did arrive, the deliveries began to be politicised, with politicians wanting to publicise that they have secured the goods.

In-charges coped with stock-outs in varying ways. One facility simply gave patients a prescription and asked them to purchase drugs from private chemists, which is a common practice in many facilities across the country when drugs are not available in the facility. In a more rural facility which is further from an urban centre, the in-charge used his/her own money to buy drugs and then sold them to patients at the market price in order to save patients the transport costs, while in another the in charge borrowed drugs from another facility to be returned upon receipt of supplies from county. In the final facility the in-charge–who was on leave–reported that other facility staff were procuring drugs and selling them to patients ‘at exorbitant prices’ to make a profit. Being on leave, (s) he was unable to take any action and upon completion of leave, she was moved to another facility in a promotional capacity.

Thirdly, although not specific to Public Primary Health Facility in-charges, salary delays for health workers across the county resulted in relationship tensions and staffing shortages at facilities. As part of devolution, human resource management for health was moved to county level, including payment of salaries. As a result of political pressure to devolve fast, this move took place suddenly in 2013 and long before structures, systems and key senior positions were in place at county level. This led to salary delays in July 2013, and significant anxiety and fear among staff at all levels, including facility in-charges. In Kilifi County, further delays and non-payment of allowances were experienced in January 2014, culminating in a circular from the Director of Health to all medical officers to require all staff to appear for a head count on 20^th^ and 21^st^ February. Staff took a day away from the facility to participate in the exercise, which included questions about counties of birth and ethnicity. These questions, in a context of wider political contestation and uncertainty, fuelled tensions and concerns among staff that ‘outsiders’ may lose their position. At all levels, managers and staff were trying to cope with a confused and shifting situation, and concerns about political motives and job insecurity. This anxiety continues to linger almost two years post devolution with occasional health worker strikes in several counties with kilifi nurses holding frequent dialogue meetings with county health managers to air their grievances. While salary debates and delays are not uncommon in Kenya, there was a heightened level of uncertainty and anxiety over this period of change.

### The key role of sub-county managers in supporting front-line workers

Over the entire observation period, facilities remained open and functioning despite repeated strains and shocks, only some of which were caused or exacerbated by devolution as it unfolded. A key support system for in-charges over this period was the sub-county managers who have played the role of line managers to in-charges in Kilifi for decades in some cases.

A sub-county manager mentioned that they encourage in-charges from the outset to turn to them if they have problems. S/he mentioned explaining to in-charges being posted to new facilities:

*…We are not posting you there to go and die suffering alone*, *so all of you I beg you*, *take my number if you have problems let me know*, *but don’t stay with your problems*, *or run away from your facility going to your home telling them that “Ah the place is so hostile”*. *Don’t do that*, *tell me and me I’ll share with the entire DHMT we will see how best to help you out of the problem*, *but don’t run away from workstations…* Male sub-county manager in Sub County two

According to MOH standards the SCHMT should conduct monthly supervision visits to all health facilities, to offer support, mentor, coach and conduct on job training (OJT) to the staff working there. This routine activity is seen by managers as an opportunity for important relationship-building with the facility staff, to encourage and identify with them to avoid isolation and demotivation. As a manager explained:

…*It is to interact with the facility staff*, *so that you can identify with them*, *any problems and possibly support them to see that they don’t continue suffering*. *Otherwise*, *others may be very discouraged; others may be very demoralised to remain in the facilities if nobody ever goes to say hi to them*. *But if you go there*, *you chat with them*. *If possible you take a drink with them*. *They feel I’m not wasted; I’m not alone…* Male sub- county manager in Sub-County two

These visits, when they happen, are appreciated by in-charges:

*…when we have challenges they can assist us with counselling*, *if we have shortages they can help us with getting some drugs here and there*, *sometimes they are also overwhelmed by events and maybe miss one quarter and come the other quarter*, *but support supervision it is well done here and we appreciate a lot…* Male PHC in-charge in facility four

Given the challenges of funding to cover all facility based supportive supervision visits, the monthly facility in-charge meetings that take place in the county headquarters are also highly appreciated. These meetings allow information to be shared, progress to be tracked, and problems and solutions to be aired and shared with peers.

Sub-county managers have sometimes worked with or had to challenge MCAs, who have both supported and challenged the in-charges in their initiatives. There were regular reports of MCAs being vocal about health workers failing to heed to free service policy for example, and some have argued that health workers are selling government drugs to patients. These complaints have sometimes been raised to the most senior county health managers who have then acted on them by bypassing the in charge’s immediate line managers.

## Discussion

PHC in-charges remain an important source of leadership and coordination of care for the majority of poor populations despite the challenges highlighted in many studies [25–29]. In this paper we contribute to a small but growing body of literature focusing on the micro-processes of governance in low income countries, and specifically some of the day-to-day roles and challenges faced by facility in-charges over a period of accelerated decentralisation in Kenya.

Overall, we show that in-charges have complex and diverse roles, which they have to perform in a difficult environment with relatively little formal leadership preparation and training. In addition to their clinical care roles, in-charges also manage clinical services, and take on more strategic tasks such as leading the development and implementation of facility Annual Operation Plans (AOPs). These roles require engagement with, and management of, a complex web of people within facilities, communities and upwards in health system hierarchies. Thus as Daire and Gilson[29] have argued, ‘*PHC facility management is not primarily a mechanistic or administrative function*, *entailing efficient implementation of predesigned roles*, *tasks and instructions*, *but is instead a dynamic and strategic process occurring in conditions of uncertainty’* (p 96).

The four health facility in-charges we worked closely with draw on different aspects of their facilities’ basic hardware to cope with crises (the everyday challenges and systems shocks),with the smaller dispensaries least able to draw on other staff members and resources and apparently having more often to resort to foregoing breaks and working beyond official working hours. Other strategies observed to cope with crises included borrowing from other facilities, purchasing drugs on behalf of patients, and increasing user fees, with such strategies sometimes developed and supported through formal decision-making structures such as FHCs and sub-county managers. These strategies illustrate the importance of tangible software such as knowledge, skills and processes of decision making in coping with system challenges; but also and perhaps even more importantly, the centrality of intangible software such as relationships, communication practices, values and norms, and intrinsic motivation in ensuring that services keep running in the face of challenges (Fig 2).

Although such strategies suggest that PHC facility in-charges at this level of health system are able to use their available resources and capacities to cope with daily challenges, there is also a potential fatigue and burn out. To sustain such strategies there is need for adequate support and maintenance over a long period. The latter has been observed by Topp et al [28] in Zambia, where the cumbersome nature of hard-copy data-collection tools and the high burden of work and the pressure to complete tasks quickly were contributing to the slow grinding down of facilities. In our observations, most facilities remained open and reasonably busy over our observation period, with staff attending to a constant stream of patients, and particularly busy periods in the mornings and on Mondays. However we were not able to measure and track utilisation rates over the time before and after removal of user fees.

In coping with daily and unusual and specific crises, our study suggests the critical importance of the sub-county managers in mediating between, translating and integrating county changes with in-charge roles and responsibilities. Although devolution has created uncertainty and confusion at the (sub) county level, mid-level managers continued to perform their roles as best as they could over the period, including through drawing on donor resources and support wherever possible to hold facility in-charge meetings and–less successfully–to continue with supervision activities. The importance of this support was regularly expressed by in-charges for enhancing motivation and providing greater job satisfaction. This reflects the findings by Elloker et al in South Africa, who in focusing on the sub district, observed the critical role of sub-district managers and their teams in galvanising front line actors to improve routines and relationships [6, 16]. Other studies in South Africa and in Ghana have illustrated the importance of a team based organisational culture and trust in management as beneficial for motivation; something that has the potential to be built upon and strengthened at a time of on-going change in Kenya. The Ghanaian study also supports the importance of good informal working relationships, a shared understanding and commitment to a mission, and a hands on and supportive management styles as protective against frustration and system level barriers [30].

Given the inter-connectedness of relationships at different levels of the health system [31], in-charges and sub-county managers are inevitably impacted upon by the broader ‘environments’ or contexts in which they are embedded. Work in South Africa [17]suggests that although conflict is a common outcome in such situations, with negative implications for health managers, providers and patients, there is also the opportunity to bring key actors together to discuss the organisational structures and processes, and identify misalignments and associated constraints. In addition, developing positive individual and organisation capabilities in an environment of stress, constraints and uncertainty requires that managers be resilient, reflective, and continuously able to learn, analyse and adapt [16]. Strategies to build and strengthen system hardware and tangible and intangible software are essential, including building managers who are able to “deal with the calculated chaos of managing–its art and craft…” by developing the managerial mind-sets–or competencies–of reflection, analysis, worldliness, collaboration and action. As Gilson and Daire [32] argue, this requires leadership development programmes that focus on generating values-based leaders that are able to manage complexity, and a continued working towards changing the system within which people work, even as they develop people as leaders.

## Conclusion

The PHC in-charges and the facilities they manage remain important to the realisation of the health of the majority population in resource poor settings. They have to contend with lack as well as multiple accountability demands. Drawing on the Ortiz Aragon framework of hardware, tangible and intangible software helped us understand how the in charge copes with scarcity as well as change. Front line health workers need to be resilient, reflective, and continuously be able to learn and adapt in such an environment. Interventions to develop and strengthen capacity at the primary public health facility level and perhaps improve performance, calls for focus not only on system hardware but also on the tangible and–possibility particularly importantly—intangible software.
